# Association of the germline *TP53 *R337H mutation with breast cancer in southern Brazil

**DOI:** 10.1186/1471-2407-8-357

**Published:** 2008-12-01

**Authors:** Juliana G Assumpção, Ana Luíza Seidinger, Maria José Mastellaro, Raul C Ribeiro, Gerard P Zambetti, Ramapriya Ganti, Kumar Srivastava, Sheila Shurtleff, Deqing Pei, Luiz Carlos Zeferino, Rozany M Dufloth, Silvia Regina Brandalise, José Andres Yunes

**Affiliations:** 1 Centro Infantil Boldrini, Campinas, Brazil; 2 Department of Oncology, St. Jude Children's Research Hospital, Memphis, USA; 3 International Outreach Program, St. Jude Children's Research Hospital, Memphis, USA; 4 Department of Biochemistry, St. Jude Children's Research Hospital, Memphis, USA; 5 Department of Pathology, St. Jude Children's Research Hospital, Memphis, USA; 6 Department of Biostatistics, St. Jude Children's Research Hospital, Memphis, USA; 7 Universidade Estadual de Campinas, Campinas, Brazil; 8 Universidade Federal de Santa Catarina, Florianópolis, Brazil

## Abstract

**Background:**

The germline *TP53*-R337H mutation is strongly associated with pediatric adrenocortical tumors (ACT) in southern Brazil; it has low penetrance and limited tissue specificity in most families and therefore is not associated with Li-Fraumeni syndrome. However, other tumor types, mainly breast cancer, have been observed in carriers of several unrelated kindreds, raising the possibility that the R337H mutation may also contribute to breast tumorigenesis in a genetic background-specific context.

**Methods:**

We conducted a case-control study to determine the prevalence of the R337H mutation by sequencing *TP*53 exon 10 in 123 women with breast cancer and 223 age- and sex-matched control subjects from southern Brazil. Fisher's test was used to compare the prevalence of the R337H.

**Results:**

The R337H mutation was found in three patients but in none of the controls (p = 0.0442). Among the carriers, two had familial history of cancer meeting the Li-Fraumeni-like criteria. Remarkably, tumors in each of these three cases underwent loss of heterozygosity by eliminating the mutant *TP53 *allele rather than the wild-type allele. Polymorphisms were identified within the *TP53 *(R72P and Ins16) and *MDM2 *(SNP309) genes that may further diminish *TP53 *tumor suppressor activity.

**Conclusion:**

These results demonstrate that the R337H mutation can significantly increase the risk of breast cancer in carriers, which likely depends on additional cooperating genetic factors. These findings are also important for understanding how low-penetrant mutant *TP53 *alleles can differentially influence tumor susceptibility.

## Introduction

Mutations in the *TP53 *tumor supressor gene usually occur within the highly conserved DNA-binding domain (aa 100–298) and, when inherited, are typically associated with Li-Fraumeni syndrome (LFS), in which carriers develop a broad spectrum of cancers (e.g., breast, brain, soft tissue, bone, blood, and adrenal cortex tumors) during childhood or young adulthood [1]. Families with incomplete features of LFS are referred to as having Li-Fraumeni-like (LFL) syndrome [2,3].

In southern Brazil, a unique germline *TP53 *point mutation resulting in an Arg to His amino acid substitution (R337H) within the C-terminal oligomerization domain is strongly associated with childhood adrenocortical tumors (ACT) [4-6]. Unlike the protypical DNA binding mutants, p53-R337H retains significant activity, although its thermal stability is reduced and it is highly sensitive to slight changes in pH [7]. The R337H mutation has a low overall malignant potential but is remarkably organ-specific, affecting mainly the adrenal cortex [4].

In most southern Brazilian families bearing the R337H mutation, pediatric ACT is the only malignancy observed [4-6]. However, in about 10–20% of the cases [[6], Seidinger et al., manuscript in preparation] the family cancer history fulfills the criteria for LFL syndrome with breast cancer being one of the most common malignancies [6]. Achatz and coworkers [8] identified R337H carriers among Brazilian LFL families, including one case of breast carcinoma that underwent loss of the wild-type allele and retained the mutant allele. These observations suggested that the R337H mutation may play a role in breast tumorigenesis. To test whether the R337H is associated with breast cancer, we retrospectively examined the prevalence of the mutation in a group of Brazilian women with breast cancer.

## Methods

### Patients and Clinical Data

The study group comprised 123 women with breast cancer that had been diagnosed and treated at the Hospital of the State University of Campinas (CAISM/UNICAMP), São Paulo, Brazil. The patient's cohort consisted of a subgroup of women who presented a familial history of breast cancer (n = 45) and another subgroup consisted of sporadic breast cancer cases (n = 78). The criteria for the selection of individuals with familial history were: more than three cases of breast cancer and more than one case of ovarian cancer in the family; more than two first-degree relatives with breast cancer; one case of male breast cancer [9,10]. Thirty of the 45 patients with positive familial history were previously tested for *BRCA1/2 *mutations [11]. A control group of 223 age-matched female volunteers was selected among university personnel. Family cancer history and blood samples were obtained with informed consent under the guidelines and approval of the Ethical Research Committees of the Faculty of Medical Sciences (FCM) at UNICAMP and The Centro Infantil Boldrini, and the National Committee of Ethics in Research (CONEP). Aditional clinical information, slides and paraffin blocks were obtained for those patients who were shown to carry the R337H mutation.

### DNA analysis

Genomic DNA was isolated from peripheral blood by using a standard phenol-chloroform extraction method [12]. Paraffin-embedded tumor samples were treated with xylene-100% ethanol (1:1) and digested with proteinase K (14 mg/mL) in 50 mmol/L Tris-HCl (pH 8.0) before phenol-chloroform extraction.

*TP53 *exon 10 was amplified by using primers and PCR conditions described by Figueiredo et al. [6], with the exception of the forward primer used for amplification of exon 10 DNA from tumor biopsies: 5'-CCATCTTTTAACTCAGGTACTG-3'. TP53 intron 3 and exon 4 were amplified by using primers 5'-CCCTAGCAGAGACCTGTGGGAA-3' (foward) and 5'-AGGCATTGAAGTCTCATGGAA-3'(reverse). *TP53 *PCR products were directly sequenced by using the BigDye terminator cycle sequencing ready reaction kit (Applied Biosystems, Foster City, CA) in an ABI PRISM 3700 automatic sequencer (Applied Biosystems). The sequences were compared to the human *TP53 *reference sequence (National Center for Biotechnology Information, accession number U94788). All mutations were confirmed by a second, independent analysis. Tumor DNA was also screened for *TP53 *alterations in other exons and introns where technically feasible, essentially as previously described [13]. In cases heterozygous for codon 72 and ins16, haplotypes were determined by performing an allele-specific PCR assay as described by Osorio et al. [14]. The *MDM2 *promoter was amplified with primers described by Bond et al. [15] and PCR products were analyzed by restriction fragment length polymorphism as described by Walsh et al. [16].

### Immunohistochemistry

Expression of p53 protein was examined by immunohistochemistry with the DO7 monoclonal antibody (Dako) at 1:100 dilution. The slides were incubated with Envision labeled polymer components (Dako, K1491) and developed with 3-3-diaminobenzidine (Sigma, D5637). Tissue from borderline ovarian tumors was included in each assay as a positive control for p53. Slides were interpreted at 40× magnification by a single pathologist. p53 protein expression was considered positive when more than 10% of the nuclei were stained; staining intensity was not considered.

### Statistical analysis

Fisher's exact test (FREQ procedures in SAS v9.1.3, SAS Institute Inc., Cary, NC) was used to compare the frequency of the R337H mutation in the breast cancer and control groups. The R337H mutation rate in the breast cancer group was estimated and the 95% exact CI reported. This rate was compared to the estimated population frequency of the mutation (assumed to be the true prevalence rate for the population) in the region of interest in southern Brazil by using the one-sided exact test for binomial proportion.

## Results

### Identification of the germline R337H *TP53 *mutation in breast cancer patients

The R337H mutation was detected in the germline DNA of three of 123 breast cancer patients in southern Brazil. None of the 223 control subjects carried the R337H mutation or any other mutation in *TP53 *exon 10. There was a significant association between the presence of the R337H mutation and breast cancer (p = 0.0442; Fisher's exact test, Table 1). The mutation rate of R337H in the cancer-positive group was 2.4%, with an exact Blyth-Still-Casella 95% CI of 0.7% to 6.6%. The overall frequency of the R337H in the region of interest in southern Brazil is assumed to be in the range 0.2%–0.3% [[17], B. Figueiredo, personal communication]. Therefore, the frequency of the R337H mutation was significantly greater among patients with breast cancer than in the general population of the region (p = 0.002 and 0.006 in comparison to a population prevalence rate of 0.2% and 0.3%, respectively; one-sided exact binomial test).

**Table 1:** Relation of the R337H *TP53 *mutation to breast cancer status

**Gene**	**Codon**	**Genotype**	**Cases (%)**	**Controls (%)**	**P value**
TP53	337	Arg/Arg	120 (97.6%)	223 (100%)	0.0442

		Arg/His	3 (2.4%)	0	

### Family cancer history

Table 2 summarizes clinical findings for the three carriers of the R337H mutation. All three developed breast cancer at an early age (19, 29, and 44 years). Patient 2 had a second cancer (brain tumor) at the age of 31 years. Patient 1 had no familial history of cancer. Patient 2 had two first-degree relatives with cancer; although the tumor type and age of onset were not known, this family might fulfill LFL-syndrome criteria. Patient 3's family cancer history met Birch's criteria [2] for LFL: a mother with bilateral breast cancer at age 53, three maternal aunts with post-menopausal breast-cancer, a sister with leukemia at age 48, and a grandson with adrenocortical carcinoma at age 18 months.

**Table 2:** Clinical findings and family cancer history of the three *TP53 *R337H carriers

**Patient**	**Tumor Type**	**Age Onset**	**TP53 IHC**	**Family Cancer History**
1	Breast; phyllodes, malignant	19 years	Negative	No cancer

2	Breast; invasive ductal carcinoma Brain tumor (malignant)	29 years 31 years	Negative Positive	Two first-degree relatives (1-U; 1-A)

3	Invasive ductal carcinoma	44 years	Positive	Six first-degree relatives (4-BC; 1-L; 1-ACC)

IHC = immunohistochemistry, U = uterus, A = arm (unknown type of cancer), BC = breast cancer, L = leukemia, ACC = adrenocortical carcinoma

### Selection against the mutant *TP53 *R337H allele during breast tumorigenesis

Tumors with a germline or somatic *TP53 *mutation usually experience loss of heterozygosity (LOH) in which the wild-type allele is deleted or mutated. Surprisingly, the mutant R337H allele was deleted in all three of the studied breast tumors. No somatic mutations were detected within sequences that could be determined (exons 2, 4, 5, 7, 8, 9, and 10) in any of the breast tumors. By contrast, the brain tumor of patient 2 was homozygous for the R337H *TP53 *mutation, it also had a second, acquired mutation (T125M), in the DNA binding domain.

### Tumor expression of p53 protein

R337H-mutant protein accumulates within the nuclei of ACT cells and is detectable by immunohistochemistry, as is other missense mutant p53 expressed in tumors [4]. Breast tumor tissue from patients 1 and 2 was negative for nuclear p53, consistent with selection against the mutant allele. However, breast tumor tissue from patient 3 expressed nuclear p53. Because this tumor had deleted the mutant allele (Table 2), this finding implies that a somatic *TP53 *mutation outside of exon 10 was acquired during tumorigenesis. However, insufficient tumor tissue hindered the identification of this genetic alteration. The brain tumor tissue from patient 2 also stained positive for nuclear p53, consistent with retention of the allele encoding 337H and acquisition of a second-site mutation (T125M) within the DNA binding domain (See additional file 1: IHC and LOH results of tumor samples from R337H patients).

### Polymorphisms associated with the R337H mutation and breast cancer

To investigate whether *TP53 *polymorphisms were associated with the R337H mutation in the three breast tumor patients, we tested for two common *TP53 *variants (R72P and ins16) reported to affect breast cancer susceptibility [18-21]. Patients 1 and 3 were heterozygous in the germline for both the R72P and ins16. Patient 2 was homozygous for R72 and negative for ins16 (Table 3). In the tumor tissue of patients 1 and 3, codon 72 encoded only arginine, indicating a selection against the proline allele.

**Table 3:** Germline and tumor genotype of *TP53 *and *MDM2*

**Patient**	**DNA Sample**	**TP53 c 337**	**TP53 c72**	**TP53 Ins16**	**TP53 Haplotypes**	**MDM2-309**
1	Blood	Arg/His	Arg/Pro	Ins+/Ins-	Arg;ins-/Pro;ins+	T/G
	Breast Tumor	Arg	Arg			

2	Blood	Arg/His	Arg/Arg	Ins-/Ins-	Arg;ins-/Arg;ins-	T/G
	Breast Tumor	Arg	Arg			
	Brain Tumor	His	Arg			

3	Blood	Arg/His	Arg/Pro	Ins+/Ins-	Arg;ins-/Pro;ins+	T/T
	Breast Tumor	Arg	Arg			

Due to early age of tumor onset, patient 1 was also tested for *BRCA1/2 *(data not shown) and a polymophic change was detected at *BRCA2 *exon 10 (N372H). The other two R337H carriers were not screened for *BRCA1/2 *mutations.

We also investigated the presence of the SNP309-G within the first intron of the *MDM2 *gene, which is a key negative regulator of p53 protein [22]. SNP309-G has been reported to accelerate tumor formation in carriers of a *TP53 *mutation [15]. Patients 1 and 2 were heterozygous for SNP309-G (G/T) and patient 3 was homozygous for the T allele (T/T) (Table 3).

## Discussion

Our findings show that the germline *TP53 *R337H mutation is associated with breast cancer in southern Brazil within the context of LFL-like families and a sporadic case. In our case-control study, three (2.4%) breast cancer patients, but none of the age-matched control subjects, carried this mutation. This frequency is approximately 10 times that estimated in the general southern Brazilian population (0.2% to 0.3%; p = 0.0221) [[17], B. Figueiredo, personal communication].

Germline *TP53 *mutations are estimated to occur in no more than 0.25% of patients with breast tumors, regardless of family history [23-25]. Therefore, the R337H mutation is the most common inherited *TP53 *mutation associated with breast cancer in southern Brazil. It is also the most frequently reported germline mutation in the International Agency for Research on Cancer database [26,27], predominantly because of its association with most pediatric ACT cases (~80%–95%) in southern Brazil [4,5]. However, the high incidence of breast cancer in this region is unlikely to be solely attributable to the R337H *TP53 *mutation, as only 2.4% of women with breast cancer in this cohort carried the mutation.

It remains unclear how the R337H mutation contributes to breast tumorigenesis. Remarkably, all three tumors selected against the mutant allele and retained the wild-type allele. In striking contrast, virtually all pediatric ACTs found to be associated with a germline R337H mutation had selected against the wild-type allele [4,5], including that of the grandson of patient 3 (data not shown). These results suggest that the mechanism of R337H-associated ACT tumorigenesis differs from that of R337H-associated breast cancer. The R337H mutation appears to play a role in accelerating the onset of breast tumorigenesis, but may not affect tumor progression in some individuals, however further studies are required to clarify this matter.

Many families that carry the germline R337H mutation are selectively predisposed to childhood ACT [4,6], although some appear to be at higher risk of more diverse cancers, including breast tumors and possibly other LF spectrum neoplasms such as brain tumors. Indeed, the R337H has been detected in LFL syndrome cases [8], but these represent the minority of the families positive for R337H [[6], Seidinger et al., manuscript in preparation]. We believe that the cancer predisposing character of low-penetrant *TP53 *mutations, such as R337H, depends on additional genetic modifiers.

Our observations of *TP53 *polymorphisms (R72P and ins16) and *MDM2 *(SNP309) are consistent with this hypothesis. Two out of the three R337H positive patients were heterozygous for the R72P and ins16 polymorphisms (Table 3). Although the consequence of these polymorphisms is controversial, proline-72 is proposed to function less well in triggering apoptosis and tumor suppression [28]. Likewise, ins16 within intron 3 diminishes *TP53 *expression [29] and has been implicated in ovarian and breast cancer [18,30,19,31]. Patients 1 and 2 who were 19 and 29 years old at diagnosis, respectively, were heterozygous for *MDM2 *SNP309 (G/T), while patient 3 who was 44 years old at diagnosis, was homozygous for the T allele (T/T). These findings are in line with previous reports showing that individuals carrying a *TP53 *germline mutation who are heterozygous or homozygous for SNP309-G develop tumors on average 10 years earlier than carriers of *TP53 *mutations who lack SNP309 [15,32]. Moreover, patient 1 also carries the N372H polymorphism at *BRCA2 *exon 10, which might contribute to breast tumorigenesis [33].

It is possible that the R337H mutation in combination with these polymorphisms and possibly other genetic alterations creates a p53-insufficient state that enhances tumor susceptibility. The degree of p53 function retained by a particular mutant and influenced by genetic modifiers within its signaling pathway dictates the level of tumor risk, which we refer to as the *TP53 *gradient effect [34].

In conclusion, we showed that the *TP53*R337H mutation is significantly associated with breast cancer in southern Brazil. However, this mutation is likely to account for only a small proportion of breast cancer cases in this region. Our results illustrate the complexity of constitutional predisposition to cancer, including that associated with *TP53 *mutations. A rigorous family history of cancer, p53 predicted functional characteristics, the presence of other genetic modifiers, and examination of the tumor cells for loss of heterozygosity must be considered in risk assessment analysis and genetic counseling.

## Abbreviations

ACT: adrenocortical tumor; LFS: Li-Fraumeni syndrome; LFL: Li-Fraumeni-like; LOH: loss of heterozygosity; SNP: single nucleotide polymorphism; BRCA1/2: breast cancer genes 1 and 2.

## Competing interests

The authors declare that they have no competing interests.

## Authors' contributions

JA wrote research project, conducted molecular genetic studies and prepared manuscript draft. AS carried out genetic molecular studies and data bank. MM, RR, GZ and SB conceived the project and revised the manuscript critically. KS and DP carried out statistical analysis. SS and RG carried out molecular studies in paraffin-embedded tumor samples. LZ and RD are medical doctors responsible for patients and IHC analysis. JY worked on research design, data analysis and manuscript drafting.

## Pre-publication history

The pre-publication history for this paper can be accessed here:



## Supplementary Material

Additional file 1 IHC and LOH results of tumor samples from R337H patients. First column shows HE staining in tumor slides from the R337H breast cancer patients, second column shows p53 immunohistochemistry in the same tumors, third column corresponds to electropherograms showing loss of heterozygosity at codon 337 in each tumor. Patient 2's brain tumor and patient 3's breast tumor showed a strong staining pattern for p53, while only patient 2's brain tumor showed LOH with retention of the 337H allele. Click here for file
