# Association of the germline TP53 R337H mutation with breast cancer in southern Brazil

**DOI:** 10.1186/1471-2407-11-152

**Published:** 2011-04-26

**Authors:** Juliana G Assumpção, Ana Luíza Seidinger, Maria José Mastellato, Raul C Ribeiro, Gerard P Zambetti, Ramapriya Ganti, Kumar Srivastava, Sheila Shurtleff, Deqing Pei, Luiz Carlos Zeferino, Rozany M Dufloth, Silvia Regina Brandalise, José Andres Yunes

**Affiliations:** 1 Centro Infantil Boldrini, Campinas, Brazil; 2 Department of Oncology, St. Jude Children's Research Hospital, Memphis, USA; 3 International Outreach Program, St. Jude Children's Research Hospital, Memphis, USA; 4 Department of Biochemistry, St. Jude Children's Research Hospital, Memphis, USA; 5 Department of Pathology, St. Jude Children's Research Hospital, Memphis, USA; 6 Department of Biostatistics, St. Jude Children's Research Hospital, Memphis, USA; 7 Universidade Estadual de Campinas, Campinas, Brazil; 8 Universidade Federal de Santa Catarina, Florianópolis, Brazil

## Correction

Of the 123 patients included in our work [1], 44 were selected according to the following familial risk history criteria: early onset (less than 35 years of age); bilateral carcinoma; more than three cases of breast cancer and more than one case of ovarian cancer in the family; more than two first degree relatives involved; and male breast cancer. We regret that in the methods section of our manuscript [1], it was erroneously stated both that the number of patients with familial history was 45 and that the criteria used for their selection was: "had either more than three cases of breast cancer and more than one case of ovarian cancer in the family; or had more than two first-degree relatives with breast cancer; or had one case of male breast cancer" (only two out of these 44 patients would have fulfilled these criteria).

Fortunately, the information regarding the familial history of our breast cancer patients was added for descriptive reasons and was not a part of any results, statistical analysis or conclusions.

## Pre-publication history

The pre-publication history for this paper can be accessed here:

http://www.biomedcentral.com/1471-2407/11/152/prepub
