# Supplementary figures and images for: Association of the germline TP53 R337H mutation with breast cancer in southern Brazil
Source: BMC Cancer. 2008 Dec 1;8:357. doi: 10.1186/1471-2407-8-357 (PMC2631588; doi:10.1186/1471-2407-8-357)

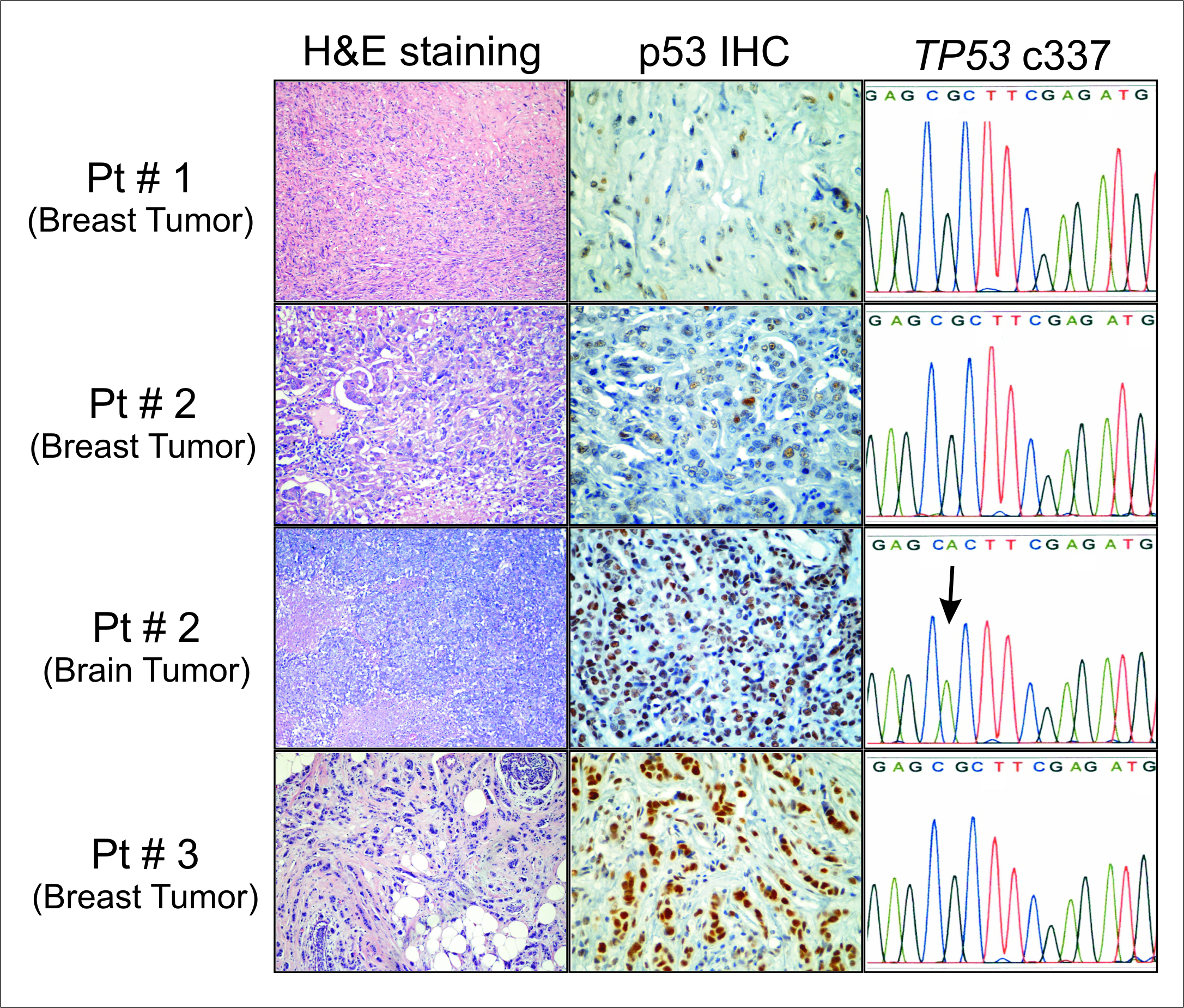

Supplement: Additional file 1 — IHC and LOH results of tumor samples from R337H patients. First column shows HE staining in tumor slides from the R337H breast cancer patients, second column shows p53 immunohistochemistry in the same tumors, third column corresponds to electropherograms showing loss of heterozygosity at codon 337 in each tumor. Patient 2's brain tumor and patient 3's breast tumor showed a strong staining pattern for p53, while only patient 2's brain tumor showed LOH with retention of the 337H allele. [file 1471-2407-8-357-S1.jpeg]
